# The Importance of the Biological Impact of Exposure to the Concept of the Exposome

**DOI:** 10.1289/EHP140

**Published:** 2016-06-03

**Authors:** Kristine K. Dennis, Scott S. Auerbach, David M. Balshaw, Yuxia Cui, Margaret Daniele Fallin, Martyn T. Smith, Avrum Spira, Susan Sumner, Gary W. Miller

**Affiliations:** 1 Department of Environmental Health, Rollins School of Public Health, Emory University, Atlanta, Georgia, USA; 2 Biomolecular Screening Branch, Division of the National Toxicology Program, and; 3 Exposure, Response, and Technology Branch, Division of Extramural Research and Training, National Institute of Environmental Health Sciences, National Institutes of Health, Department of Health and Human Resources, Research Triangle Park, North Carolina, USA; 4 Department of Mental Health, Bloomberg School of Public Health, Johns Hopkins University, Baltimore, Maryland, USA; 5 Division of Environmental Health Sciences, School of Public Health, University of California, Berkeley, Berkeley, California, USA; 6 Division of Computational Biomedicine, School of Medicine, Boston University, Boston, Massachusetts, USA; 7 Discovery Sciences, RTI International, Research Triangle Park, North Carolina, USA

## Abstract

**Background:**

The term “exposome” was originally coined in 2005 and defined as the totality of exposures throughout the lifetime. The exposome provides an excellent scientific framework for studying human health and disease. Recently, it has been suggested that how exposures affect our biology and how our bodies respond to such exposures should be part of the exposome.

**Objectives:**

The authors describe the biological impact of the exposome and outline many of the targets and processes that can be assessed as part of a comprehensive analysis of the exposome.

**Discussion:**

The processes that occur downstream from the initial interactions with exogenous and endogenous compounds determine the biological impact of exposures. If the effects are not considered in the same context as the exposures, it will be difficult to determine cause and effect. The exposome and biology are interactive—changes in biology due to the environment change one’s vulnerability to subsequent exposures. Additionally, highly resilient individuals are able to withstand environmental exposures with minimal effects to their health. We expect that the vast majority of exposures are transient, and chemicals underlying exposures that occurred weeks, months, or years ago are long gone from the body. However, these past chemical exposures often leave molecular fingerprints that may be able to provide information on these past exposures.

**Conclusions:**

Through linking exposures to specific biological responses, exposome research could serve to improve understanding of the mechanistic connections between exposures and health to help mitigate adverse health outcomes across the lifespan.

**Citation:**

Dennis KK, Auerbach SS, Balshaw DM, Cui Y, Fallin MD, Smith MT, Spira A, Sumner S, Miller GW. 2016. The importance of the biological impact of exposure to the concept of the exposome. Environ Health Perspect 124:1504–1510; http://dx.doi.org/10.1289/EHP140

## Introduction

A wide variety of initiatives and approaches, such as the Human Genome Project, Genome-wide association studies (GWAS), whole genome sequencing, and the Encyclopedia of DNA Elements (ENCODE) project, have highlighted the importance of genomics in health and disease. Over the past few decades, efforts to understand environmental contributors have not been as robust. Research has clearly established that the environment plays a significant role in our health and in the development of disease, and comprehensive studies of genetic variants and disease have been conducted to reveal links between environmental exposures and health outcomes (Doll and Peto 1981; Remington and Brownson 2011). For example, a recent meta-analysis of heritability of human traits from more than 10,000,000 twin pairs determined that 49% of the variation was genetic in origin (Polderman et al. 2015) with up to 51% of the variation potentially associated with the environment. While the human traits studied are distinct from disease vulnerability, such attributes do contribute to the overall health state of an individual. Other studies have identified environmental factors as significant contributors to disease, yet the specific exposures of concern are poorly defined. The need remains for a concerted and organized effort to systematically evaluate environmental contributors to health and disease (Lichtenstein et al. 2000; Willett 2002). Christopher Wild coined the term exposome and defined it as the totality of our exposures from conception onward (Wild 2005); Miller and Jones (2014) refined this definition to include “the cumulative measure of environmental influences and associated biological responses throughout the lifespan.” Exposures come from our external environment and lifestyle (e.g., diet, stress, smoking, chemicals, drugs, microbes) and are also the result of our internal biological processes and metabolism that generate new biological intermediates (Rappaport and Smith 2010). Through understanding the internal processes in the context of external exposures, interventions can be made at both the individual and societal level to mitigate health risks (Smith et al. 2015). The study of the exposome provides the opportunity for the research community to develop and apply existing tools that allow a comprehensive evaluation of environmental factors that can be used in coordination with efforts to study genetic factors in health and disease (Cui et al. 2016). Complementary tools and approaches to study both the genetic and environmental factors that contribute to disease have the potential to revolutionize biomedical science.

From a human health perspective, we are mostly concerned with those exposures that are associated with adverse health outcomes. Thus, it is not the presence of the chemicals per se that is the concern, but how those chemicals are altering our biology. Such effects could include binding to macromolecules, inducing structural changes (e.g., DNA mutation, adducts, epigenetic modifications) and disruption of enzyme function, or damage via reactive oxygen or nitrogen species. Our bodies also possess a myriad of processes that work to mitigate the effects of the aforementioned actions. A complex DNA repair system works to correct the vast majority of DNA damage that occurs on a daily basis. Ubiquitination, autophagy, and proteolysis help process damaged proteins to allow for recycling of the building blocks. Therefore, the net effect of a particular exposure is the sum of the adverse effects from the insult and the body’s attempt to repair or respond to the insult. One can define the former as the biological effect and the latter as the biological response. Together, the effect and response can be defined as the biological impact. The biological impact thus results from the exposure and ongoing attempt of the body to remain in a state of homeostasis. Through teasing out the proportion of the biological impact due to non-genetic influences, the cumulative cost of this correction process could provide an important measure of the exposome.

Indeed, the level of resilience within an organism is key to maintaining health. Individuals who exhibit a high degree of resilience are able to withstand environmental insults with minimal effects to their health. The ability to respond in a resilient manner also impacts the biology of an individual. The biological impact could be considered to be outside the concept of the exposome if one takes the view that the exposome is exclusively focused on the chemical exposures and processes. However, if one views the exposome as an overarching paradigm for health and disease, the biological impact becomes a component of the exposome itself (Miller and Jones 2014).

Biomarkers of exposure, dose, response, and susceptibility were defined between 1987 and 1989 (NRC 1987), and have been extended and summarized by others (Sumner and Fennell 2007). In this paradigm, biomarkers are viewed on a continuum between markers of exposure and markers of effect, with markers of susceptibility spanning the continuum. The traditional definition being that a biomarker of exposure is a chemical, its transformation, reaction, or degradation product, or an adduct to a macromolecule derived from these chemicals or products. Markers of biological effect are established, for example, through comparison of case and control, target and non-target tissues, or dose- and time-to-response, where a correlation between the biomarker and biological effect can be demonstrated. Markers of susceptibility are commonly defined as the genetic factors that influence the body’s sensitivity to a chemical but can also include biological factors such as age, nutritional status, and lifestyle; these markers help capture an individual’s overall resilience to exposures. Markers of susceptibility are revealed through study designs using for example, sensitive and nonsensitive species, transgenic models or more recently, gene–environment interaction studies on a GWAS/environment-wide association study (EWAS) scale (Patel et al. 2013). Significant progress continues to be made in using molecular and analytical methods to measure biomarkers for linking exposure with health outcomes, and new approaches in exposome research will enable a more comprehensive and integrated analysis across the biomarker continuum (Dennis et al. 2016).

Exogenous chemicals can cause thousands of perturbations to our bodies. However, from a health standpoint, we are most concerned with those effects that are most likely to disrupt our health. It is rather amazing that faced with altered temperature, activity, energy uptake, and psychological challenges, we can maintain a rather consistent blood pressure, weight, and body temperature. These key functions operate under a series of cooperative homeostatic mechanisms that sense alterations and respond in a way to minimize the change in the system. However, the goal of these systems is not always to return the system to exactly where it was before the challenge. This process of dynamic homeostasis has been termed allostasis, with the concept of allostatic load representing the cost of the cumulative correcting process. By capturing the wear and tear process on our bodies, allostatic load may provide a clinically relevant means of measuring the biological response as it relates to the exposome. The concept of allostatic load has been cultivated within the stress research community (McEwen 1998; McEwen and Wingfield 2003). As defined, it may not provide exactly what exposome research needs, but it could be a model for a derivation that does provide the right metrics for exposome research. For example, telomere length and the epigenetic aging clock may be useful indices for assessing long-term wear and tear (Mitchell et al. 2014; Needham et al. 2013; Ornish et al. 2013).

Cumulative lifetime exposures ultimately impact health and disease outcomes but many of these exposures are short-lived and leave no long-term chemical trace in the body. The biological response provides a means of identifying transient exposures through the molecular fingerprints left on the body. Changes such as telomere length, epigenetic modifications, and protein adducts may be able to provide a window into past exposures where no other measurement is available. Additionally, a purported challenge for the exposome is capturing life-course exposures. Measuring everything all the time is a daunting task but in reality, this is not necessary. Snapshots in time where one sample can measure both surrogates of past exposures and current chemicals and their metabolites in the body make measuring the exposome a more achievable task. The specific targets (Table 1) that mediate the biological effects and response represent the subjects of investigation. Through our discussion, we will address the strategies for measuring the biological impact, what these measurements offer for exposome research and the overall advancement of health, and what initial steps are currently underway to measure the biological response.

**Table 1:** Range of biologically relevant targets as they relate to exposome research.

Target site	Example health implications (e.g.)	Media for measurement
Epigenetic
Methylation	Colorectal Cancer risk Severity of fragile X syndrome	Cord blood, maternal plasma (fetal DNA), placental tissues, other tissue samples, whole blood, plasma, buffy coat, dried blood spots, cell culture
Hydroxymethylation (5hmC)	Cancer risk due to hypomethylation of oncogenes	Cord blood, maternal plasma (fetal DNA), placental tissues, other tissue samples, whole blood, plasma, buffy coat, dried blood spots, cell culture
Oxidized nucleotides	Blocking DNA repair pathways	Cord blood, maternal plasma (fetal DNA), placental tissues, tissue samples, whole blood, plasma, buffy coat, dried blood spots, cell culture
Histone modification	Role in alcohol-induced liver disease	Cord blood, maternal plasma (fetal DNA), placental tissues, tissue samples, whole blood, plasma, buffy coat, dried blood spots, cell culture
RNA-associated silencing (e.g., antisense transcripts, noncoding RNAs, RNA interference)	RNA interference involved in T-cell acute lymphoblastic leukaemia	Cord blood, placental tissues, tissue samples, whole blood, plasma, buffy coat, dried blood spots, cell culture
Receptors
Activation	Selective activation of the vitamin D receptor in chronic kidney disease can reduce oxidative stress and inflammation, increasing positive outcomes	Cell culture, ligand binding assays
Inhibition	M-methyl-D-aspartate receptor (NMDAR) inhibition by lead exposure impacts synapse development	Cell culture, ligand binding assays
DNA damage
Mutations	Haemophilia, cystic fibrosis, phenylketonuria, etc.	Cord blood, placental tissues, tissue samples, whole blood, plasma, buffy coat, dried blood spots, cell culture
Adducts	Increased mutagenic and carcinogenic risk	Cord blood, placental tissues, tissue samples, whole blood, plasma, buffy coat, dried blood spots, cell culture
Chromosome
Sequence variant	Causal factor in Mendelian disorders Risk factor in complex disorders Susceptibility factor for biological response to exposure	Any tissue, and multiple solutions (EDTA, etc.) acceptable (except maybe formalin fixed); timing of collection not critical
Copy number	Down syndrome (duplicated chromosome) Huntington’s disease (repeated triplet sequence) Autism (small-large duplications and deletions)	Any tissue, and multiple solutions (EDTA, etc.) acceptable (except maybe formalin fixed); timing of collection not critical
Structural abnormality	Smith-Magenis syndrome	Cord blood, placental tissues, tissue samples, whole blood, plasma, buffy coat, dried blood spots
Micronuclei frequency	Indicator of environmental exposure to genotoxic agents and cancer risk	Cord blood, tissue samples, whole blood, plasma, buffy coat, dried blood spots, cell culture
Microbiome
Metabolites	Microbial pathways involved in production of short chain fatty acids (SCFAs): SCFAs have been implicated in obesity and diabetes	Whole blood, plasma, buffy coat, urine, cell culture
Diversity/composition	Third trimester gut microbiome composition can induce greater insulin insensitivity, increasing the risk of gestational diabetes	Stool, swab samples
Proteins
Post-translational modifications	Changes to mitochondrial proteins play a role in tissue injury in alcoholic and nonalcoholic fatty liver disease	Cord blood, placental tissues, tissue samples, whole blood, plasma, buffy coat, cell culture
Translational regulation	Hyperferritinaemia or cataracts disease due to excessive production of the iron-storage protein ferritin	Cord blood, placental tissues, tissue samples, whole blood, plasma, buffy coat, cell culture
Regulatory RNA species
miRNA	High levels of certain miRNA expression linked to Parkinson’s disease pathogenesis; potential tool for better diagnosis and therapy	Cord blood, placental tissues, tissue samples, whole blood, plasma, buffy coat, cell culture
siRNA	siRNA as treatment for Huntington’s disease	Cord blood, placental tissues, tissue samples, whole blood, plasma, buffy coat, cell culture
mRNA	Increased BACE1 mRNA expression in Alzheimer’s disease	Cord blood, placental tissues, tissue samples, whole blood, plasma, buffy coat, cell culture

## Discussion

### Assessing Biological Impact

From a scientific perspective, we must consider the realistic ways in which we can assess the biological impact of exposures. Here, we discuss the potential sites from which to draw samples and review the technologies available to assess various biological alterations. Biological fluids (i.e., blood, serum, plasma) and excreta (i.e., saliva, feces, urine) collected with minimally invasive methods are ideal for sampling in clinical and epidemiological investigations aimed at understanding how the environment perturbs molecular, microbial, and biochemical pathways. Multiple sample types across relevant matrices for the various analytical methods need to be employed to arrive at the coverage of exposures and their impacts. Analytical methods have commonly been used to measure a wide range of parent compounds (e.g., metals, phthalates, minerals, drugs, chemicals) and their related metabolites. Just as important, questionnaires and other data collection techniques have been utilized to determine other sources of exposure, including indicators of stress, anxiety, or mental health that may also impact health. These biological specimens and other data collection instruments can be utilized to help reveal how exposures have altered the overall physiological and psychological status over time. Past examples include the use of traditional methods to determine the link between exposure and the formation of DNA adducts and health impact. Newer approaches are now being utilized: for example, the metabolomics of urine or microbial analysis of feces as methods to evaluate the metabotype (i.e., the overall biochemistry) or microbial populations of an individual at any point in time, and to determine how those are related to measurements of exposure. Revealing which factors (e.g., metabolites, proteins, microbial populations, DNA adducts, cytochrome P450s) are most critical for defining the state of disease or dysfunction, and the correlation with factors of exposure, will provide a means to develop intervention strategies.

With the increasing availability of omics technologies and continually developing database resources such as the Kyoto Encyclopedia of Genes and Genomes database, perturbations to the molecular system can be identified to show disturbed pathways that may be indicative of disease (Kanehisa and Goto 2000). While investigations with samples derived from epidemiological and clinical studies will provide us with biomarkers and mechanistic insights through pathway mapping, it is important to correlate these findings in other tissues. In some cases, this will be accomplished through biopsy samples, where measurement in biological fluids, excreta, and other tissues can be compared. However, it is also critical to obtain these types of samples from dose- and time-to-response studies using experimental model systems to gain detailed mechanistic information. Dose–response studies in animal, cellular, and tissue models facilitate the comparison of subtle alterations at lower concentrations with the more robust changes seen at higher doses. These laboratory models can reveal targets and susceptibility factors through concentration–response studies. As low-dose exposures are often difficult to identify in human subject investigations due to matrix effects (i.e., unexpected reduction or enhancement of the analyte response due to other components in the sample), this provides a way to determine low-dose marker profiles to validate in human subject investigations and discern previously unknown exposures and their related effects.

### Technologies

With the increasing availability of high-throughput techniques, the revolution of omics-based approaches, and enhanced capabilities in bioinformatics and mathematical modeling, we are positioned to simultaneously capture and model information for a wide range of exposures, and how these exposures individually and cumulatively correlate with the impact on biological pathways (Vineis et al. 2009; Vlaanderen et al. 2010). The biological responses from exposure create the physiological state of an individual at any given time and define the conditions under which a new exposure could initiate disease. Table 2 highlights some of the major technologies that are currently being deployed in exposome research with a focus on those that assess aspects of the biological impact. It is expected that significant advances in these and new technologies will continue as exposome research moves forward. Scientists and researchers who are exploring new technologies in this field would be wise to not commit to any particular approach at this point, but rather keep abreast of technological developments and continually test and adopt those approaches that most accurately assess the biological impact of exposures. However, some technologies are primed for integration into exposome studies and should be prioritized in current research. Although it is beyond the scope of this article, it is worth noting that the continued development of bioinformatics approaches and databases to capture the massive amounts of data currently being generated will be key to linking exposures to biological responses and disease outcomes. Bioinformatic needs for exposome research were addressed by a separate working group and will be highlighted elsewhere. Here we describe some of the current technologies available and propose guidelines for choosing the most relevant assay and a few recommendations of tools that serve as starting points to measure biological effects.

**Table 2:** Major technologies that are currently being deployed in exposome research.

Approaches	What it measures	Specific technique	Coverage of “ome”	Throughput (low, medium, high)
Metabolomics	Metabolite signals, typically of > 10,000 endogenous and exogenous metabolites	NMR spectroscopy	Unknown, not all metabolites mapped yet	High
		Chromatography-Mass spectrometry	Unknown, not all metabolites mapped yet	Low to medium
Epigenomics	DNA methylation	Illumina MethylationEPIC Bead Chip 850K DNA methylation array	Promoters, CpG islands, shores, open sea that has previously shown variability across tissues or disease states	Medium to high
		Reduced Representation Bisulfite Sequencing (RRBS)	Restricts sequencing to areas of genome with high CpG content	Medium to high
		Whole-genome bisulfite sequencing	Full coverage of genome	Low to medium
	Histone modifications	ChIP-seq	Coverage of whole genome across most cell types	High
Adductomics	Macromolecules covalently bound to endogenous macromolecules like DNA and protein	High-resolution mass spectrometry	Allows detection of both known and unknown adducts	High
Proteomics	Post-translational changes to proteins at the cellular level	Soft ionization mass spectroscopy	Less targeted approach that allows capture of unknown proteins and protein complexes	Low to medium
		Antibody microarrays	Protein expression coverage based on probes available	Low
Transcriptomics	Nucleotide-level resolution of RNA expression	Hybridization-based technologies	Identification of any sequences included in array/technique	High
		RNA-seq	Full coverage of any RNA sequence in sample of interest, including low abundance transcripts	Medium
Genomics	Sequences and examines functions of genes	Next Generation Sequencing	Full coverage of genome	Medium to high
High-throughput screening	Receptor activity (e.g., estrogen, androgen, aryl hydrocarbon, G-protein signaling, ion channel activation)	Chemical Activated Luciferase gene eXpression (CALUX)	Selected receptors across wide range of media	High
		High content analysis	Phenotyping across many cell-based assays	Medium

***Technologies in common clinical use***. A number of technologies are currently available that will allow for biological phenotyping and determination of physiological state from the sample types described above. More traditional end points such as clinical chemistries and hematological assessments are clinically informative, highly automated, and inexpensive. Further, there are a variety of clinical tests that are used less routinely that provide greater depth of characterization of a biological state including measurement of endocrine hormones and enzyme assays indicative of specific disease states. For example, accepted clinical practices for diagnosing Cushing’s syndrome, a disorder of the adrenal glands, take a standard approach with recommendations for an initial screening test and further validation through additional tests (Nieman et al. 2008). Although important for treatment, a diagnostic approach to diseases through these methods does not allow for the identification of upstream targets in the biological response through which a preventive intervention could occur. A large, yet incomplete list of these tests is available from providers [e.g., LabCorp (https://www.labcorp.com/wps/portal/testmenu/)]. The challenge with these less routinely used tests is that they are typically low throughput and quite costly. Hence, alternative means of determining physiological state using omics-based approaches would be ideal. Along these lines, there has been success in characterizing different molecular biological domains using samples obtained through minimally invasive procedures. Although records of these clinical measures can be useful in establishing disease state or selection of a research population, these measures should be combined with more comprehensive indicators of biological impact, such as epigenomics, DNA adduct formation, cytokine panels, or metabolomics in efforts to understand the exposome.

***Genomics***. As mentioned, genetic predisposition to biological impacts of the exposome is a critical aspect of understanding the effect on health and disease. While whole-genome sequencing is now possible, the price, storage, and computational efforts that are necessary preclude this as a main source of genomics data for epidemiological studies. Instead, many single nucleotide polymorphisms (SNPs)–based arrays are now available that can query anywhere from 500,000 to 5M SNPs at a time. Data from these genome-scale measurement tools can be used to “impute” genotypes across the genome for most ancestral populations, assuming the proper match of array design and population. While these are now standard and relatively affordable, several specialized subset arrays are now available for a much lower cost and greater scalability. These query genetic variation in only the subset of genes in the genome related to a particular mechanism, such as a “metabochip” that measures SNPs in genes known to code metabolically relevant proteins or an “immunochip” that covers immune-related genes. Characterization of the genetic background provides a baseline from which to understand gene–environment interactions or environmentally related biological perturbations.

***Epigenomics***. Epigenetic marks, such as DNA methylation and histone modifications, are known to be modifiable by exogenous and endogenous environments (Ho et al. 2012). The majority of epidemiologically useful epigenetic measurements consider DNA methylation, although other epigenetic marks such as histone modifications and chromatin structure are possible to measure when large amounts of fresh tissue are available. For DNA methylation, several genome-scale assays are currently available that query methylation at specific CpGs across the genome. Some are highly biased towards promoters, while others include non-promoter and inter-genetic regions that have been previously shown to harbor differences in methylation between tissue types or disease states and are thus potentially biologically relevant in populations. The most affordable and commonly used of these assays is the Illumina Infinium 450K DNA methylation array that queries over 450,000 CpGs as well as non-CpG SNPs from which DNA barcoding and ancestry estimation are possible. The recently released Illumina Methylation EPIC BeadChip offers an 850K DNA methylation array, allowing increased throughput while minimizing cost. High-throughput full genome sequencing approaches to measure DNA methylation are also dropping in price and surely on the horizon for epidemiologic scale in the future, but currently face the same constraints as whole-genome sequencing mentioned above. Reduced representation bisulfate sequencing (RRBS) offers another less costly alternative to full genome methylation profiling with 2 million sites across a representative sample of the human genome.

More notable findings that suggest epigenomics may be helpful in characterization of biological state include signatures associated with “molecular age,” heart disease, cancer, chronic inflammation, diabetes, and a number of other chronic diseases (Ghantous et al. 2015). Research indicates that epigenomics has particular utility for picking up on early-life exposures occurring in key developmental windows. A historic example is the Dutch Hunger Winter cohort where offspring of undernourished pregnant mothers had different epigenetic profiles than did siblings born before or after that period and increased risks of certain conditions (Heijmans et al. 2008; Lumey et al. 2007). Environmental exposures have demonstrated impact on epigenetic signatures and development of diseases such as cancer (Jaenisch and Bird 2003). A long-term vision for exposome research would be cataloguing the epigenetic impact of various classes of chemicals. This could ultimately facilitate our understanding on which transient, short-term exposures have occurred that other biomarkers cannot currently pick up.

***Transcriptomics***. Chemical exposure can drive changes in RNA expression through activation of signaling pathways. Such chemical-associated changes in expression have been characterized quite extensively in animal models and human *in vitro* systems. A few studies have investigated biological states in blood using transcriptomics while others have used transcriptomics to characterize the biological state of the microbiome. In one study on the impact of *in utero* exposure to arsenic, transcript profiles indicated potential long-term health impacts on immune function for the exposed infants (Fry et al. 2007). Although analytical challenges remain, gene-expression profiling offers a window into estimating the genetic and environmental influences on transcription and downstream disease processes (Gibson 2008). Two technologies for transcriptomics are primarily used; hybridization-based microarrays are the first generation technologies and a second generation technology called RNA-seq employs next-generation sequencing technologies to characterize the transcriptome at nucleotide-level resolution.

***Proteomics***. Exposure to environmental chemicals often elicits a change in cellular signaling which is carried out in part by changes in the post-translational state of proteins (e.g., ubiquitin, myristoylation, glycosylation, phosphorylation, nitrosylation, acetylation, methylation, hydroxylation). Further, impacting these signaling pathways by chemical exposure gives rise to changes in protein abundance that are secondary to changes in the transcriptome. The global effects on the proteins of the cell are referred to as proteomics. A number of omic-scale technologies have emerged in recent years that provide quantitative and qualitative characterization of the proteome. Two basic approaches have emerged in proteomics that are based on soft ionization mass spectroscopy and antibody microarrays. Proteomics technologies have been previously used to evaluate serum, saliva, feces and the microbiome. Depending on the disease process of interest, proteomics may be a useful indicator of the biological impact of exposure.

***Adductomics***. Reactive chemicals will often covalently bind to endogenous macromolecules such as DNA and protein. Adduction of these macromolecules has the potential to alter their function leading to alterations in the biological state of a cell, tissue or organism. Due to the long half-life of some of these adducts they have been used as an indicator of chemical exposure (Rappaport et al. 2012). Detecting DNA adducts in a given tissue can be suggestive of an individual experiencing a high level of exposure, the body’s inability to properly respond to an exposure or some combination of both (Poirier et al. 2000). The technologies that are employed in adductomics are similar to those used in metabolomics and proteomics (Balbo et al. 2014). Blood adductomics has been employed extensively in exposure assessment however adductomics approaches using saliva samples are also employed to evaluate chemical DNA binding. Due to the health implications of DNA and protein adducts, adductomics is one technology with immediate utility for measuring biological effects.

***Metabolomics***. Metabolomics involves the study of the low molecular weight complement of cells, tissues, and biological fluids and excreta. Nuclear magnetic resonance (NMR) spectroscopy, chromatography, and mass spectrometry methods are used to detect signals for metabolites that define the metabolome. Additionally, researchers recently demonstrated the reliable quantification of chemicals through high-resolution metabolomics (Go et al. 2015). The Human Metabolome DataBase (HMDB) provides information on 10,000 endogenous and exogenous metabolites, their pathways, and their potential relevance to disease (Wishart et al. 2007, 2013). There are a variety of subdomains of metabolomics that focus on characterization of different metabolite domains including lipidomics and glycomics. Metabolomics also provides a means to assess pathways impacted by different microbial species (16S rRNA sequencing) and functional categories (metagenomic sequencing) of microbial diversity present. Given the broad coverage metabolomics provides, it is primed for utilization in large population-based studies characterizing exposures and particularly for measuring biological impact (Athersuch 2016; Su et al. 2014). Metabolomics lends itself to broad characterization of disease pathways, allowing for assessment of pathway perturbations that may be an early indicator of initial disease processes. An eventual outcome of this understanding would be the ability to alter that process early enough to change the individual’s health trajectory.

***Functional assays***. A variety of functional assays are on the market that can help assess cytotoxicity, apoptosis, DNA repair capacity, etc. across various instrument platforms. High content screening assays are one example of a relatively new tool with high throughput potential. High content screening allows for the development of specific cellular assays to screen a range of chemical compounds for toxicity indicators. Due to the versatility of this platform, multiple cellular endpoints can be targeted such as neurite length, neuronal morphology and cell viability for specific neuron types. Chemically Activated LUciferase gene eXpression, or CALUX, bioassays are highly sensitive and reliable high throughput screenings to test receptor activity in various mediums including soil, drinking water and serum or plasma from human blood. The activation of various receptors can be analyzed including the estrogen, androgen, glucocorticoid and aryl hydrocarbon (Ah) receptors in human or mammalian cells (Brouwers et al. 2011; He et al. 2011). Similarly, high-content imaging can be employed to measure a host of receptor-based activities, from receptor translation, G-protein signaling, to ion channel activation.

The Tox21 initiative through the National Institutes of Environmental Health Sciences (NIEHS) is an example of the utility of these screening tools for the assessment of thousands of chemicals across multiple assays (Tice et al. 2013).

### Guidelines for Assay Selection

Of the listed technologies in Tables 1 and 2 and those described above, the question remains how does one choose the most relevant assays to assess biological impact in exposome research. Although this should continue to be an evolving conversation, there are a few questions that can be used as guiding principles:

Is there a disease outcome of interest and what is known about the pathology? Assay selection can be guided by known mechanisms or pathways of a particular disease in order to better assess the potential role of environmental influences. If disease pathology is well-characterized for a particular tissue this may facilitate the development and use of a new assay for screening environmental exposures and disease pathways. Although the impact of complex exposures on disease pathways is poorly understood, the wealth of information available regarding disease processes should be utilized to maximize our ability to characterize the biological response.
For the outcome of concern, is there a suspected influence? For example, reproductive and developmental outcomes may be more likely to have an epigenetic component.
Are there known biomarkers already measured in the population of interest and does this inform what further screening should be done? If the prevalence of a particular inflammatory biomarker or chemical, for example, is high in a population subgroup, this can direct screening for related biological markers or suggest functional assays for a particular class of chemicals or target tissues.
Is there not much known about the complex exposures within a population? If yes, then an approach such as metabolomics would allow for pathway analysis and general screening to identify leads for further inquiry.

### Current Directions for Measuring the Biological Response

One of the challenges with assessing biological impact is determining which alterations are due to particular exposures or combinations of exposures. Integrating biological response measures with questionnaire data or real-time exposure monitoring tools will help elucidate the exposures linked with a particular biological impact. Approaches that are amenable to pathway analysis and network construction can help create models that are grounded in biology. Such approaches allow convergence of data from multiple platforms. Cross-omics informatics tools are being developed and investigators interested in the exposome should draw upon these efforts to develop models that can communicate with other datasets (Vlaanderen et al. 2010). For example, if new exposome-based networks can build upon existing frameworks used in epigenetics or proteomics, once exposure-related data is acquired it can be readily integrated into existing models of health and disease. Additional challenges remain in determining the biological impact of exposures across time (e.g. chronic, sporadic or single time point exposures). Through integrating biological response measures with other exposure assessment tools such as real-time monitoring devices and questionnaire approaches, we can better understand exposure patterns and variability in biological response across time.

A recent NIEHS initiative will serve to promote and facilitate the use of established biological response markers in children’s environmental health research. The Children’s Health Exposure Analysis Resource (CHEAR) serves as a structure to facilitate evaluation of the early-life exposome (NIEHS 2015). In addition to a coordinating center and data repository, analysis and science center, CHEAR has six laboratory hubs that provide consultative and analytical services across targeted, untargeted, and biological response research cores. Developmental cores within the hubs offer an opportunity to explore and validate emerging techniques and markers of biological impact, including omics approaches for pathway analysis.

The biological response resource cores offer a wide range of expertise. A few highlights include assessment of epigenetic changes, gene expression, redox status, DNA damage, immune response, psychosocial stress, and mitochondrial function (NIEHS 2015). Although each core in the hubs functions independently, the hubs are highly integrated with opportunities to link data across resources. As CHEAR progresses, there will naturally be growth in the biological response resource cores as new markers are validated. The synergy across CHEAR offers the potential to advance techniques for measuring and understanding the early-life exposome.

## Recommendations

The exposome is continuing to gain traction within the research community. By attempting to capture the impact of complex environmental exposures, the concept of the exposome could be expanded. Specific patterns of exposures could be linked to specific biological responses such that historical patterns of exposure may be assessed. These responses encompass the initial interaction of compounds with molecular targets as well as the alteration of pathways and systems.

The following recommendations are suggested to advance exposome-related research as it relates to biological response:

**Recommendation 1**—Include the biological impact of exposures in analyses of the exposome. Aspects of biological response have not always been included in the concept of the exposome but are essential to identify which exposures are biologically important.
**Recommendation 2**—Biological effects should be evaluated from a systems- and pathways-based perspective rather than focusing on singular biological targets. This will require high-throughput approaches and advanced bioinformatic solutions as noted in recommendation 4.
**Recommendation 3**—Demand reasonable and biologically feasible explanations of contributors to disease. One of the advantages of the exposome approach is that one has access to biological data, exposure data, and health outcomes. This makes it possible to go beyond simple associations to more complex models that include the biological components important to understanding causal pathways.
**Recommendation 4**—Develop bioinformatics approaches that link exposures with biological responses and disease outcomes. The exposome will be represented as multifactorial variables. This will require sophisticated informatics approaches.
**Recommendation 5**—Develop databases or coordinating centers including establishing language standards for exposures. The massive amount of data and complex unifying models will require collaborative data storage, access, and analysis. This could take the form of open-source programs and tools combined with controlled data archiving.
**Recommendation 6**—Provide guidelines for sample collection standards for use with emerging and anticipated technologies. Samples collected for traditional exposure assessment may be inconsistent with the needs of measuring biological response. Guidelines for exposome research must encompass the needs of all potential users.
**Recommendation 7**—Develop criteria for selecting the best assay(s) to assess biological response for the research question of interest. These criteria should be updated periodically to address emerging tools and technologies.

## Conclusions

By anchoring exposures to specific biological alterations, the exposome can link exposures to health outcomes and also provide guidance into testing the biological plausibility of exposures in laboratory models. While classical epidemiological studies identify associations between exposures and health outcomes, they often require additional support on biological mechanisms and plausibility in order to make statements about probable cause. By incorporating biological responses into the exposome, biological plausibility is built into the framework. Thus, the inclusion of the biological response in exposome research will help close the gap between association and causation in environmental health research by providing testable mechanistic connections between exposure and disease that integrate environment and lifestyle data with ongoing genomic, proteomic, and metabolomic initiatives. Furthermore, it helps ground the exposome in well-documented and testable biological models. One critique of the exposome is that it is impossible to measure all exposures all the time but biological responses of past exposures represent a level of memory that reduces the need to capture historical exposure data. These specific biological alterations will help guide future research, clinical treatment, and public health strategies aimed at mitigating adverse effects.
